# The Effect of Social-Emotional Competency on Child Development in Western China

**DOI:** 10.3389/fpsyg.2019.01282

**Published:** 2019-06-07

**Authors:** Yehui Wang, Zhaoxi Yang, Yingbin Zhang, Faming Wang, Tour Liu, Tao Xin

**Affiliations:** ^1^Collaborative Innovation Center of Assessment for Basic Education Quality, Beijing Normal University, Beijing, China; ^2^Curriculum and Instruction, University of Illinois at Urbana-Champaign, Champaign, IL, United States; ^3^Key Research Base of Humanities and Social Sciences of the Ministry of Education, Academy of Psychology and Behavior, Tianjin Normal University, Tianjin, China; ^4^Faculty of Psychology, Tianjin Normal University, Tianjin, China; ^5^Center of Collaborative Innovation for Assessment and Promotion of Mental Health, Tianjin, China

**Keywords:** social-emotional competency, social-emotional learning (SEL), academic achievement, academic emotions and attitudes, interpersonal relationship

## Abstract

This study investigated the effects of social-emotional competency on pupils’ academic achievement, academic emotions and attitudes, and interpersonal relationships. Participants were 7106 fourth-grade and fifth-grade students in western China. The results were: (1) social-emotional competency positively predicted pupils’ academic achievement (including reading, mathematics, and science); (2) social-emotional competency predicted pupils’ academic emotions and attitudes, including learning anxiety and interest, and academic emotions and attitudes played a mediating role in the relation between social-emotional competency and academic achievement; and (3) social-emotional competency positively predicted pupils’ interpersonal relationships, including peer relationships and teacher–student relationships, and interpersonal relationships played a mediating role in the relation between social-emotional competency and academic achievement. These findings highlighted the importance of social-emotional competency to child development in western China, where many children might lack their parents’ company.

## Introduction

In 1994, Goleman et al. proposed the concept of social-emotional learning (SEL) and set up Collaborative for Academic Social, and Emotional Learning (Collaborative for Academic Social, and Emotional Learning [CASEL], 2018). SEL refers to the process through which “children and adults acquire and effectively apply the knowledge, attitudes, and skills necessary to understand and manage emotions, set and achieve positive goals, feel and show empathy for others, establish and maintain positive relationships, and make responsible decisions” (Collaborative for Academic Social, and Emotional Learning [CASEL], 2018). It aims to enhance students’ core social-emotional competencies (SEC), including self-awareness, self-management, social awareness, relationship skills and responsible decision-making (DePaoli et al., 2017). With the improvement of SEC, SEL programs could lead to measurable and potentially long-lasting improvements in many areas of children’s lives (Greenberg et al., 2017).

Numerous studies have proven the positive impact of SEC on academic performance. For example, students’ SEC at the beginning of an academic year could predict their grades atthe end of the academic year (Elias and Haynes, 2008). A meta-analysis based on 213 SEL programs showed that, on average, 11% of the increment in academic scores was because of the programs (Durlak et al., 2011).

Studies have found that SEC can affect students’ academic emotions and attitudes. Zins et al. (2007) argued that once students possess social-emotional skills, they can apply them to increase their academic engagement, thereby also increasing their motivation to learn. Carrying out an SEL program or enhancing children’s SEC could decrease learning anxiety (Horowitz and Garber, 2006; Wang et al., 2016). At the same time, learning interest and anxiety had significant effects on students’ academic achievement (e.g., Flowerday and Shell, 2015; Tosto et al., 2016; Skaalvik, 2018). Therefore, SEC may exert an influence on academic achievement through students’ academic emotions and attitudes.

Many studies have shown evidence that SEC positively predicted interpersonal relationships, including peer relationships and teacher–student relationships. Socially and emotionally skilled students could successfully regulate their emotions. Effective control over anger could help adolescents reconcile the differences of opinion between themselves and their friends, thus benefitting the maintenance of reciprocal peer relationships (Asher et al., 1998). In addition, children with high SEC might be more likely to have a strong connection with peers, which could increase the probability of positive peer influence (Hawley et al., 2002). These children also engaged more in teaching activities and tended to ask teachers for help when they have difficulty in learning, and thus their relationships with teachers improved and became closer (Zins et al., 2004). Moreover, SEC could indirectly influence academic performance via peer relationships and teacher–student relationships (Zins et al., 2004; DeLay et al., 2016).

Many studies have revealed that high SEC may contribute to children’s healthy development (e.g., Durlak et al., 2011; Jones et al., 2017), especially for disadvantaged students (Baker, 1999; Reyes et al., 2000). Baker (1999) and Reyes et al. (2000) claimed SEL program could help students from low-income families or ethnic minorities to get better academic achievement by raising their SEC. In China, there is a group of disadvantaged children called left-behind children whose parents work far away from home for higher salaries and leave them at home. In 2016, the number of left-behind children in rural areas of western China was approximately 3.52 million (Ministry of Civil Affairs of the People’s Republic of China, 2016). Most of them received little psychological support from their parents (Giles et al., 2010). Without parents’ company, these children might suffer from various problems, such as emotional and behavioral problems, poor academic performance and social maladjustment (Luo et al., 2009). Given that SEC can benefit other disadvantaged students, this positive impact of SEC may also exist in left-behind children. However, there is a lack of direct evidence about this benefit to convince stakeholders to conduct SEL programs to mitigate the undesirable effect of the scarcity of educational resources and parents’ companionship. Therefore, the present study aimed to provide such evidence.

**FIGURE 1:**
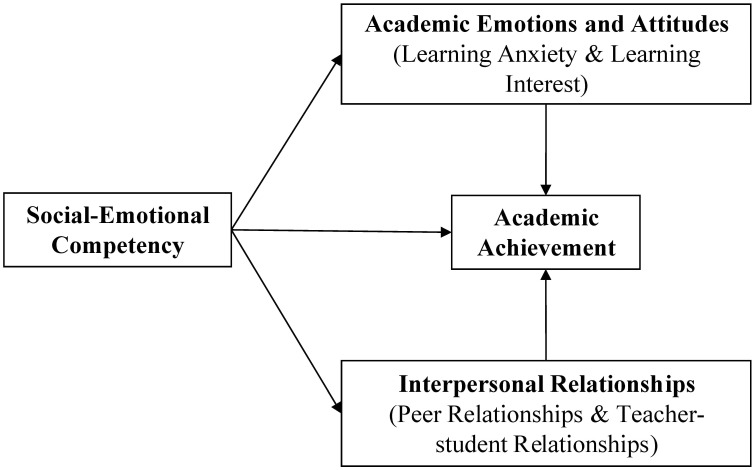
The hypothetical model of this study.

This paper examines the effects of SEC on pupils’ academic achievement, academic emotions and attitudes, and interpersonal relationships in western China. There are three hypotheses. Hypothesis 1: high SEC is associated with better pupils’ academic achievement, including reading, mathematics, and science. Hypothesis 2: high SEC is associated with better pupils’ academic emotions and attitudes, including higher learning interest and lower learning anxiety, and learning interest and anxiety mediate the association between SEC and academic achievement. Hypothesis 3: high SEC is associated with better interpersonal relationships, including peer relationships and teacher–student relationships, and these relationships mediate the association between SEC and academic achievement. Figure 1 displays the full hypothetical model.

## Materials and Methods

### Participants and Procedure

We used a stratified random sampling method to recruit 7106 students from 97 primary schools in western China. The sample comprised 3774 boys (53.1%) and 3332 girls (46.9%) from grade four (*n* = 3706, 52.2%) and grade five (*n* = 3400, 47.8%). The mean age was 11.25 (*SD* = 0.96).

With the assistance of the teachers, the researchers read the instructions, and students completed these questionnaires and tests in paper and pencil. Students took approximately 40 min to complete the background questionnaire, reading, mathematics and science tests, respectively.

### Measures

#### Social-Emotional Competency

Based on the Delaware Social and Emotional Competency Scale (Mantz et al., 2016), Zhu (2016) developed the Chinese version of the Delaware Social-Emotional Competency Scale (DSECS-SCV). The DSECS-SCV consists of 12 items, with 3 items assessing each of the four factors: responsible decision-making, relationship skills, self-management, and social awareness. Students rated each item on a 4-point Likert-type scale (1 = *not like me at all*, 2 = *not much like me*, 3 = *somewhat like me*, and 4 = *very much like me*). The scale showed good internal consistency and goodness-of-fit of confirmatory factor analysis (CFA) in the current study (α = 0.81, *χ*^2^/*df* = 10.3, *CFI* = 0.97, *TLI* = 0.96, *RMSEA* = 0.04). Table 1 displays the means and standard deviations of the items.

**Table 1:** Means and standard deviations of 12 items of social-emotional competency.

		Means	SD
Responsible	Item 1	3.37	0.88
decision-making	Item 2	3.06	0.92
	Item 3	2.87	0.95
Social awareness	Item 4	3.02	0.90
	Item 5	3.05	0.92
	Item 6	2.81	0.99
Self-management	Item 7	3.06	0.94
	Item 8	3.03	0.93
	Item 9	2.96	0.99
Relationships skills	Item 10	2.52	1.03
	Item 11	3.30	0.87
	Item 12	3.44	0.88


#### Academic Achievement

Teachers and experts in educational measurement collaboratively developed the reading, mathematics and science achievement tests in accordance with the curriculum standards (Ministry of Education of the People’s Republic of China, 2011a,b,c).

The reading achievement test consisted of 27 items and two subscales: word mastery (5 items) and text reading (22 items). The S-CVI/AVE was 0.90, and Cronbach’s alpha was 0.81. The average item difficulty coefficient was 0.51, and the average item discrimination coefficient (point-biserial coefficient) was 0.34.

The mathematics achievement test contained 30 multiple-choice items, involving knowledge about numbers and algebra, space and shape, change and relationships, uncertainty, and quantity. The S-CVI/AVE was 0.93, and Cronbach’s alpha was 0.83. The average item difficulty coefficient was 0.60, and the average item discrimination coefficient was 0.34.

The science achievement test contained 31 choice items, involving knowledge about geography, biology, physics, and environmental issues. The S-CVI/AVE was 0.91, and Cronbach’s alpha was 0.82. The average item difficulty coefficient was 0.59, and the average item discrimination coefficient was 0.33.

#### Academic Emotions and Attitudes

Measures of academic emotions and attitudes included reading interest, mathematics interest and anxiety, and science interest. Table 2 presents the features of these measures in the current study. The original scales were translated into Chinese, and the wording was modified to make them suitable for students in grade four and five in western China. Mathematics anxiety has attracted the attention of many educational researchers and practitioners, and numerous studies have revealed its negative impact on learning. Therefore, the current research investigated the link between SEC and mathematics anxiety.

**Table 2:** The scale of emotions and attitudes.

Variables	Source	Numbers	Scoring method	Cronbach’s α	Composite reliability (CR)	Average variance extracted (AVE)	McDonalds’ omegas	The goodness-of-fit indices			
								*χ*^2^/*df*	CFI	TLI	RMSEA
Reading interest	PIRLS2011	4		0.70	0.81	0.52	0.69	21.78	0.99	0.94	0.05
Mathematics interest	PISA2012	4	4-point Likert-type scale (1 = strongly disagree, 4 = strongly agree)	0.81	0.88	0.64	0.81	11.29	1.00	1.00	0.04
Science interest	PISA2015	3		0.75	0.86	0.67	0.76	0.00	1.00	1.00	0.00
Mathematics anxiety	PISA2012	5		0.81	0.87	0.58	0.82	14.65	0.99	0.99	0.04


#### Peer Relationships

Asher and Dodge (1986) proposed the peer-nomination method to measure peer relationships. During the survey, each child had a list of classmates’ names and was asked to choose three favorite classmates and three classmates whom they liked least. Then, the counts of a student being liked and being disliked by classmates were his or her liking (L) and disliking (D) scores. L and D scores were standardized within each class to get standardized L and D scores. The standardized L score minus the standard D score was the indicator of social preference (SP), while the sum of the standardized L and D scores was the indicator of social impact (SI; Asher and Dodge, 1986).

#### Teacher–Student Relationships

The teacher–student relationships in this study included reading, mathematics and science teacher–student relationships. Each variable was measured by 5 items rated on a 4-point Likert-type scale from 1 = *strongly disagree* to 4 = *strongly agree*. Table 3 presented the features of these variables. These items were adopted and translated from the teacher–student relationship scale in PISA 2012 (Organization for Economic Co-operation, and Development [OECD], 2014). The wording was modified to make these items suitable for fourth-grade and fifth-grade students in western China.

**Table 3:** The scale of teacher–student relationships.

Variables	Cronbach’s α	Composite reliability (CR)	Average variance extracted (AVE)	McDonalds’ omegas	The goodness-of-fit indices			
					*χ*^2^/*df*	CFI	TLI	RMSEA
Reading teacher–student relationships	0.84	0.88	0.61	0.84	26.01	0.99	0.98	0.06
Mathematics teacher–student relationships	0.84	0.89	0.61	0.84	31.99	0.99	0.98	0.07
Science teacher–student relationships	0.85	0.90	0.63	0.86	30.77	1.00	0.99	0.07


### Data Analyses

The descriptive analysis and correlation analysis were conducted within SPSS 23.0 as the preliminary analysis. In this step, the values of SEC, all kinds of interests and teacher–student relationships, as well as mathematics anxiety, were the mean of corresponding item scores. Structural equation modeling (SEM) using MPLUS 7.11 was conducted to test the hypothetical model and estimate the direct and indirect effects of SEC on other variables. Gender, grade, race and the left-behind state were used as control variables. For the goodness-of-fit test, the indices checked were chi-square (*χ*^2^), the comparative fit index (CFI), Tucker-Lewis index (TLI) and root mean square error of approximation (RMSEA). A *χ*^2^/*df* < 2 indicates a good fit of the hypothetical model (Hu and Bentler, 1999). The values of CFI and TLI over 0.90 are considered as accepted indices, and those over 0.95 and the value of RMSEA below 0.06 indicate a good model fit. To assess the mediational effect, a bootstrapping mediation test with 3000 bootstrapped resamples was performed, and 95% bias-corrected intervals were calculated. If zero is not in the intervals of an indirect effect, it can be concluded that this indirect effect exists at a significance level 0.05 (Preacher and Hayes, 2008).

## Results

### Descriptive Statistics and Correlations

Table 4 displays the means, standard deviations, and correlations of variables. SEC was positively related to all academic achievement, all learning interest, all teacher–student relationships, social preference, and social impact and was negatively related to mathematics anxiety. Reading academic achievement was significantly positively related to reading interest, social preference, social impact, and reading teacher–student relationship. Mathematics academic achievement was significantly positively related to mathematics interest, social preference, social impact, and mathematics teacher–student relationship. A significant negative correlation was found between mathematics academic achievement and mathematics anxiety. Science academic achievement was significantly positively related to science interest, social preference and reading teacher–student relationship. No significant relationship was between science academic achievement and social impact.

**Table 4:** Descriptive statistics and correlations of variables.

	1	2	3	4	5	6	7	8	9	10	11	12	13
1. Social-emotional competency	1												
2. Reading academic achievement	0.33***	1											
3. Mathematics academic achievement	0.32***	0.67***	1										
4. Science academic achievement	0.29***	0.62***	0.63***	1									
5. Reading interest	0.58***	0.32***	0.29***	0.26***	1								
6. Mathematics interest	0.41***	0.19***	0.25***	0.18***	0.42***	1							
7. Science interest	0.43***	0.21***	0.21***	0.24***	0.44***	0.41***	1						
8. Mathematics anxiety	–0.19***	–0.25***	–0.27***	–0.23***	–0.16***	–0.26***	–0.15***	1					
9. Social preference	0.14***	0.23**	0.20***	0.20***	0.12***	0.10***	0.07***	–0.08***	1				
10. Social impact	0.05**	0.07***	0.06***	0.02	0.03*	0.05**	0.01	–0.05**	0.11***	1			
11. Reading teacher–student relationship	0.41***	0.22***	0.22**	0.19***	0.41***	0.34***	0.40***	–0.16***	0.12***	0.05**	1		
12. Mathematics teacher–student relationship	0.41***	0.22***	0.24***	0.18***	0.40***	0.49***	0.43***	–0.20***	0.12***	0.05**	0.69***	1	
13. Science teacher–student relationship	0.37***	0.15***	0.16***	0.15***	0.38***	0.38***	0.51***	–0.15***	0.08***	0.01	0.64***	0.71***	1
*M*	3.04	500.00	500.00	500.00	3.15	3.15	3.17	2.14	0.04	–0.16	3.24	3.23	3.14
SD	0.53	100.00	100.00	100.00	0.68	0.74	0.77	0.80	1.38	1.20	0.73	0.72	0.78


*^∗^p < 0.05, ^∗∗^*p* < 0.01, ^∗∗∗^*p* < 0.001. The values of variable 1, 5–8, and 11–13 were the mean of corresponding item scores.*

### Structural Equation Modeling

**FIGURE 2:**
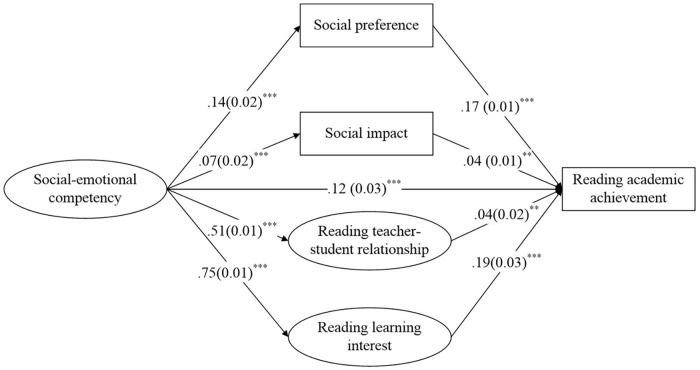
Standardized path estimates for reading. ^∗∗^*p* < 0.01, ^∗∗∗^*p* < 0.001. Circles denote latent variables and squares denote observed variables. Standardized regression coefficients (standard errors) are presented. For simplicity, confounding variables including gender, grade, race, and the left-behind state are not presented in the figure, and the standardized factor loadings of items are presented in Supplementary Tables 1–3.

Figure 2 presents the standardized path estimates of reading. Although the chi square was significant (*χ*^2^ = 2578.73, *df* = 317, *χ*^2^/*df* = 8.13), the remaining goodness-of-fit indices showed an accepted or good fit of the data, with CFI and TLI of 0.94 and 0.93, respectively (above 0.90; Hu and Bentler, 1999), and RMSEA of 0.03 (below 0.06; Hu and Bentler, 1999). The model explained 23.8% of the variance in reading academic achievement. The direct influence of SEC on reading academic achievement was significant (β = 0.12, *p* < 0.001). SEC positively predicted social preference, social impact, reading teacher–student relationship and reading interest (β = 0.14, *p* < 0.001; β = 0.07, *p* < 0.001; β = 0.51, *p* < 0.001; β = 0.75, *p* < 0.001), which had a positive effect on reading academic achievement (β = 0.17, *p* < 0.001; β = 0.04, *p* < 0.01; β = 0.04, *p* < 0.01; β = 0.19, *p* < 0.001). Thus, these variables might play mediating roles in the relation between SEC and reading academic achievement.

**FIGURE 3:**
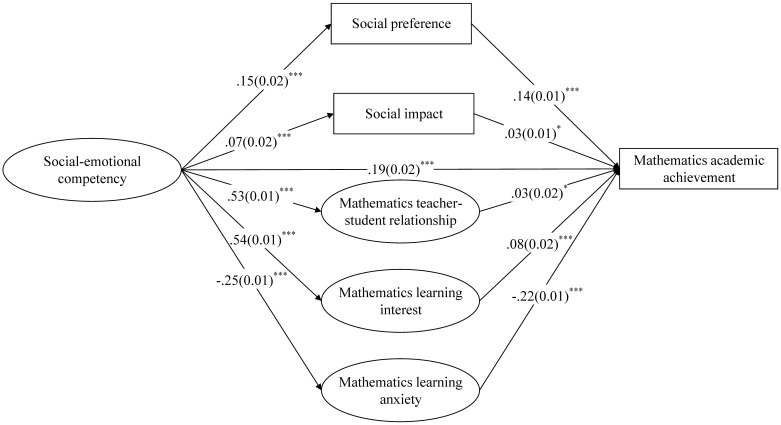
Standardized path estimates for mathematics. ^∗^*p* < 0.05, ^∗∗∗^*p* < 0.001. Circles denote latent variables and squares denote observed variables. Standardized regression coefficients (standard errors) are presented. For simplicity, confounding variables including gender, grade, race, and the left-behind state are not presented in the figure, and the standardized factor loadings of items are presented in Supplementary Tables 1–3.

Figure 3 shows the mathematics model. Although the chi square was significant (*χ*^2^ = 3630.45, *df* = 455, *χ*^2^/*df* = 7.98), the remaining goodness-of-fit indices showed an accepted or good fit of the data, with CFI and TLI of 0.94 and 0.94, respectively (above 0.90), and RMSEA of 0.03 (below 0.06). The model explained 28.4% of the variance in mathematics academic achievement. The direct influence of SEC on mathematics academic achievement was significant (β = 0.19, *p* < 0.001). SEC positively predicted social preference, social impact, mathematics teacher–student relationship and mathematics interest (β = 0.15, *p* < 0.001; β = 0.07, *p* < 0.001; β = 0.53, *p* < 0.001; β = 0.54, *p* < 0.001), and the above four variables had a significant positive effect on mathematics academic achievement (β = 0.14, *p* < 0.001; β = 0.03, *p* < 0.05; β = 0.03, *p* < 0.05; β = 0.08, *p* < 0.001). SEC was negatively related to mathematics anxiety (β = -0.25, *p* < 0.001), which negatively predicted mathematics academic achievement (β = -0.22, *p* < 0.001). Thus, social preference and impact, mathematics teacher–student relationship, mathematics interest and mathematics anxiety might serve as mediators in the influence of SEC on mathematics academic achievement.

**FIGURE 4:**
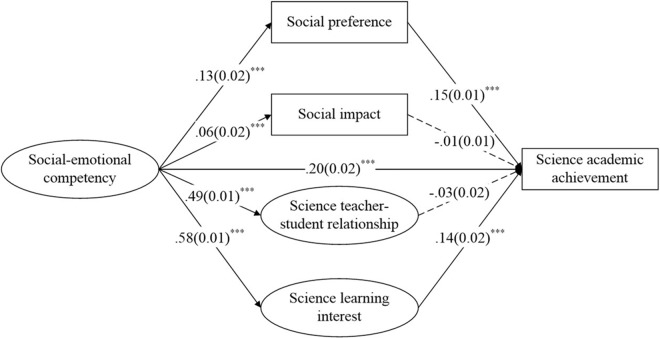
Standardized path estimates for science. ^∗∗∗^*p* < 0.001. Circles denote latent variables and squares denote observed variables. Standardized regression coefficients (standard errors) are presented. For simplicity, confounding variables including gender, grade, race, and the left-behind state are not presented in the figure, and the standardized factor loadings of items are presented in Supplementary Tables 1–3.

Figure 4 depicts the science model. Although the chi square was significant (*χ*^2^ = 3037.91, *df* = 291, *χ*^2^/*df* = 10.44), the remaining goodness-of-fit indices showed an accepted or good fit of the data, with CFI and TLI of 0.93 and 0.92, respectively (above 0.90), and RMSEA was 0.04 (below 0.06). The model explained 17.3% of the variance in mathematics academic achievement. The direct influence of SEC on science academic achievement was significant (β = 0.16, *p* < 0.001). SEC positively predicted social preference, social impact, science teacher–student relationship and science interest (β = 0.13, *p* < 0.001; β = 0.06, *p* < 0.001; β = 0.49, *p* < 0.001; β = 0.58, *p* < 0.001), and social preference and science interest had a significant positive effect on science academic achievement (β = 0.15, *p* < 0.001 and β = 0.14, *p* < 0.001, respectively). However, social impact and science teacher–student relationship did not predict science academic achievement (β = 0.00, *p* > 0.05 and β = -0.03, *p* > 0.05, respectively). Thus, social preference and science interest might play mediating roles in the relation between SEC and students’ mathematics academic achievement, but social impact and science teacher–student relationship did not.

Table 5 presents the indirect effects and their 95% bias-corrected confidence intervals. The indirect effects of SEC on reading academic achievement via social preference, social impact, reading teacher–student relationship and reading interest were significant. The indirect effects of SEC on mathematics academic achievement via social preference, mathematics teacher–student relationship, mathematics interest and mathematics anxiety were significant, while social impact’s indirect effect was not significant. For science, the indirect effects of SEC on student’s science academic achievement via social preference and science interest were significant, while the indirect effects of social impact and science teacher–student relationship were not significant.

**Table 5:** Indirect effects and 95% confidence intervals.

Indirect effects		Estimate (standardized)	95% CI
Reading	SEC → social preference → reading academic achievement	0.024	[0.017,0.031]
	SEC → social impact → reading academic achievement	0.003	[0.001,0.005]
	SEC → reading teacher–student relationship → reading academic achievement	0.020	[0.005,0.034]
	SEC → reading interest → reading academic achievement	0.143	[0.105,0.181]
Mathematics	SEC → social preference → mathematics academic achievement	0.020	[0.014,0.026]
	SEC → social impact → mathematics academic achievement	0.002	[0.000,0.004]
	SEC → mathematics teacher–student relationship → mathematics academic achievement	0.017	[0.001,0.033]
	SEC → mathematics interest → mathematics academic achievement	0.045	[0.028,0.062]
	SEC → mathematics anxiety → mathematics academic achievement	0.055	[0.046,0.064]
Science	SEC → social preference → science academic achievement	0.020	[0.014,0.026]
	SEC → social impact → science academic achievement	0.000	[–0.002,0.001]
	SEC → science teacher–student relationship → science academic achievement	–0.015	[–0.031,0.000]
	SEC → science interest → science academic achievement	0.083	[0.061,0.105]


As shown in Figure 3, social impact had a small positive effect on mathematics academic achievement (β = 0.03, *p* < 0.05). However, zero was in the confidence intervals of the indirect effect of social impact in the influence of SEC on mathematics achievement (β = 0.002, CI [0.000, 0.003]).

## Discussion

Consistent with our hypotheses, the results indicated that SEC could significantly predict pupils’ academic achievement, academic emotions, and attitudes and interpersonal relationships. SEC also exerted an indirect effect on academic achievement via academic emotions and attitudes and interpersonal relationships.

There are several unique features of the present study. First, the study showed that SEC was related to not only pupils’ academic achievement but also their academic emotions and attitudes and interpersonal relationships. This finding indicates that the effects of SEC on child development may be comprehensive. Second, this study covered three subjects (reading, mathematics, and science), and thus could further strengthen the links between SEC and pupils’ academic achievement, academic emotions and attitudes and interpersonal relationships. Finally, this study was conducted in western China, where many students are left-behind children. Parents’ migration in this area has exerted negative impacts on their children’s lives, such as little tutoring in children’s study and inability to meet children’s needs for parental affection (Ye and Pan, 2011). This study implies that enhancing children’s SEC may alleviate the problems caused by parents’ migration.

### The Effect of Social-Emotional Competency on Academic Achievement

The present study found that SEC could directly predict academic achievement when controlling for gender and grade levels. The results are in line with previous research (Durlak et al., 2011). Students with high SEC are good at setting and working toward their academic goals. When they encounter difficulties and failure, they may be better able to control their emotions and solve problems. As a result, they can overcome difficulties in learning and gain new knowledge. The direct effect of SEC on academic performance, in this study, existed in all three subjects including reading, mathematics, and science. The results revealed that the effect of SEC on performance might be consistent across subjects rather than confined to any specific subject.

### The Effect of Social-Emotional Competency on Academic Emotions and Attitudes

#### Academic Emotions and Attitudes

In the current study, SEC could also predict the learning interests of the three subjects. The results were consistent with prior research (Durlak and Wells, 1997). Students with high SEC tended to perform more self-regulation and make responsible decisions. Therefore, these students might have a deeper understanding of the meaning of learning and might not tire of studying due to the dislike of their teachers or rebelliousness. Accordingly, they would be interested in studying and able to maintain this interest.

A high level of SEC was also associated with low mathematics anxiety. This relation might be because students with high SEC were able to regulate their emotions effectively when facing academic stress, and thus, they would suffer from less anxiety, while students with low SEC regulated emotions poorly and could not successfully deal with stress, which would make them more distressed.

#### The Mediational Role of Academic Emotions and Attitudes

The learning interest and mathematics anxiety mediated the relationship between SEC and academic achievement. The learning interest could give rise to sustained attention and engagement, thereby affecting learning results (Anderson et al., 1984). This interest could also elicit spontaneous attention and free up cognitive resources, thereby benefitting more consistent cognitive representation and learning (Hidi, 1990). Students with high SEC had high learning interest and would put more effort and time into academic study. Consequently, they would earn better grades.

Mathematics anxiety had a negative effect on academic achievement. Nonetheless, the improvement of SEC could reduce students’ mathematics anxiety and thus promote mathematics academic achievement. A high level of mathematics anxiety could cause students to avoid math and could disrupt cognitive processing by compromising ongoing activity in working memory (Ashcraft, 2002). Students with low SEC might have difficulty in coping with stress and feel more math-anxious, thus performing poorly in math.

### The Effect of Social-Emotional Competency on Interpersonal Relationships

#### Interpersonal Relationships

SEC predicted peer relationships and teacher–student relationships. This finding supported hypothesis 3 and was in keeping with prior research (Brown and Conroy, 2011; DeLay et al., 2016). Students with high SEC usually master some relationship skills, such as the ability to communicate clearly, listen well and negotiate conflict constructively, which can help them maintain interpersonal relationships. A good SEL program can deepen social bonding and create a more encompassing classroom environment where students feel accepted, supported, and valued (Pianta et al., 2003; Greenberg et al., 2015). Besides, students with high SEC also tended to seek and offer help when needed. This tendency could facilitate the relationship-building between students with good and poor scores (DeLay et al., 2016).

#### The Mediational Role of Interpersonal Relationships

Peer relationships and teacher–student relationships mediated the relationship of SEC and academic achievement. An indicator of peer relationships, social preference, played a mediational role in all three subjects. High social preference means popularity. If a student is popular in the class, he or she may feel comfortable and confident. In such a situation, they are able to collaborate with others more effectively when facing challenging learning tasks (Wentzel and Watkins, 2002). Furthermore, social impact, another indicator of peer relationships, mediated the association of SEC and reading scores but not the association of SEC and mathematics or science scores. The reason for this finding might be that students with a high social impact score had better sociability and cognitive ability (Newcomb et al., 1993). They might use language more frequently than students with low social impact scores, and the reading assessment mainly tested the language ability.

In line with prior research (Zins et al., 2004), teacher–student relationships also served as a mediator in the influence of SEC on achievement in the model of reading and mathematics. High-SEC students got along well with their teachers and thus tended to engage in teaching activities and were more likely to ask for and receive help from teachers. However, it must be noted that the effect of teacher–student relationships on achievement was small. What was unexpected is that science teacher–student relationship did not mediate the association between SEC and science score because science teacher–student relationship was not correlated with science score. One possible reason for this result was that western China lacks qualified elementary teachers, especially science teachers because science is not a major subject in the traditional elementary education system in China. Only 8% of the sampled classes in the present study were taught by full-time science teachers. The science courses of the left classes were taught by reading or mathematics teachers. These part-time science teachers were unable to give students enough support and help in science because of insufficient science pedagogical knowledge, although students might get along well with them.

### Implications for Education

This study revealed that SEC could effectively promote pupils’ academic emotions and attitudes. The cultivation of academic emotions and attitudes is one of the three-dimensional objectives of curriculums in Chinese elementary and secondary education, and the Chinese government and educational practitioners have paid increasing attention to the development of pupils’ academic emotions and attitudes in recent years. However, teachers and educational practitioners are often unable to accomplish this goal in classrooms due to the lack of available methods. Based on the results of this study, the problem may be solved by fostering students’ SEC. Moreover, this study also revealed that SEC had a significantly positive effect on academic achievements, and a part of this effect was attributed to positive academic emotions and attitudes and interpersonal relationships. In the long term, children with higher SEC are more likely to be ready for college, succeed in their careers, have positive relationships and better mental health, and become engaged citizens (Greenberg et al., 2017). Overall, the effects of SEC on child development can help students succeed in the school, career, and life.

Some effective SEL programs have been carried out in various regions and have proven to be effective, such as the Promoting Alternative Thinking Strategies (Humphrey et al., 2016) and Second Step (e.g., Low et al., 2016; Gregory and Amy, 2018) programs. However, SEL programs are few in China and have only been implemented in a few regions and provinces. In the future, offering more SEL programs or infusing social-emotional skills into the regular academic curriculum should be conducted in order to help children to develop comprehensively. In addition, enhancing children’s SEC may alleviate the undesirable effects of parents’ migration, especially for left-behind children in western China who lack educational resources and parents’ companionship.

### Limitations and Outlooks

The first limitation of this research was that it was unable to infer causal relationships due to the cross-sectional research design. Longitudinal studies should be conducted to further examine the effect of SEC on academic development and interpersonal relationships. Second, students’ SEC was measured through a self-report questionnaire. The self-report results might be confounded by social desirability and students’ self-evaluation ability. It may increase the data accuracy to collect and integrate SEC data from multiple channels, such as students, parents, and teachers, rather than only from students. Besides, given that many sampled schools lacked adequate computer facilities, all questionnaires and tests were done in paper and pencil. In the future, data may be collected by computer-based assessment, which is a more flexible and secure way to ensure the data accuracy and the absence of errors. Finally, the influence of social impact and reading teacher–student relationship on reading achievement and the influence of mathematics teacher–student relationship on mathematics achievement were small. It requires more studies to examine these effects.

## Ethics Statement

The study and protocol were reviewed and approved by the Institutional Review Board of the Collaborative Innovation Center of Assessment for Basic Education Quality, Beijing Normal University. Written informed consent was obtained from the students as well as from their parents/legal guardians. All the participation was entirely voluntary.

## Author Contributions

YW contributed to developing the theoretical framework, editing of the manuscript, providing critical revisions, organization of the manuscript, and the overall design. ZY contributed to designing tools, interpreting the data, analyses, and overall writing of the manuscript. YZ contributed to designing tools, data collection, data analyses, editing of the manuscript and providing critical revisions. FW contributed to designing tools, data collection and providing critical revisions. TL contributed to data collection and providing critical revisions. TX contributed to developing the theoretical framework, designing tools and providing critical revisions. All authors approved the final version of the manuscript for submission.

## Conflict of Interest Statement

The authors declare that the research was conducted in the absence of any commercial or financial relationships that could be construed as a potential conflict of interest.
