# Supplementary material for: The Effect of Social-Emotional Competency on Child Development in Western China
Source: Front Psychol. 2019 Jun 7;10:1282. doi: 10.3389/fpsyg.2019.01282 (PMC6566918; doi:10.3389/fpsyg.2019.01282)
Supplement: Supplementary file 1 [file Table_1.docx]

# Appendixes

Table 1. The Delaware Social and Emotional Competency Scale (Mantz et al., 2016)

| Dimension (loading in SEC) | Item | Loading |
| --- | --- | --- |
| Responsible decision-making (1.00) | 1. I blame others when I’m in trouble. | 0.16 |
|  | 2. I feel responsible for how I act. | 0.58 |
|  | 3. I am good at deciding right from wrong. | 0.59 |
| Social awareness (0.91) | 4. I think about how others feel. | 0.65 |
|  | 5. I care about how others feel. | 0.66 |
|  | 6. What others think is important to me. | 0.55 |
| Self-management (0.98) | 7. I can control how I behave. | 0.58 |
|  | 8. I think before I act. | 0.61 |
|  | 9. I am good at waiting for what I want. | 0.49 |
| Relationship skills (0.89) | 10. I am good at solving conflicts with others. | 0.43 |
|  | 11. I get along well with others. | 0.64 |
|  | 12. I have one or more close friends. | 0.52 |
