# Supplementary material for: The Effect of Social-Emotional Competency on Child Development in Western China
Source: Front Psychol. 2019 Jun 7;10:1282. doi: 10.3389/fpsyg.2019.01282 (PMC6566918; doi:10.3389/fpsyg.2019.01282)
Supplement: Supplementary file 2 [file Table_2.docx]

# Appendixes

Table 2. Items of academic emotions and attitudes

| Scale | Item | Loading |
| --- | --- | --- |
| Reading interest | 1. I like talking about what I read with other people. | 0.52 |
|  | 2. I would be happy if someone gave me a book as a present. | 0.50 |
|  | 3. I would like to have more time for reading. | 0.67 |
|  | 4. I enjoy reading. | 0.71 |
| Mathematics interest | 1. I look forward to my mathematics lessons. | 0.77 |
|  | 2. I enjoy reading about mathematics. | 0.68 |
|  | 3. I do mathematics because I enjoy it. | 0.77 |
|  | 4. I am interested in the things I learn in mathematics. | 0.66 |
| Mathematics anxiety | 1. I get very nervous doing mathematics problems. | 0.71 |
|  | 2. I get very tense when I have to do mathematics homework. | 0.73 |
|  | 3. I often worry that it will be difficult for me in mathematics classes. | 0.73 |
|  | 4. I feel helpless when doing a mathematics problem. | 0.71 |
|  | 5. I worry that I will get poor <grades> in mathematics. | 0.51 |
| Science interest | 1. I enjoy learning science. | 0.74 |
|  | 2. I learn many interesting things in science. | 0.63 |
|  | 3. I like science. | 0.77 |
