# Supplementary material for: The Effect of Social-Emotional Competency on Child Development in Western China
Source: Front Psychol. 2019 Jun 7;10:1282. doi: 10.3389/fpsyg.2019.01282 (PMC6566918; doi:10.3389/fpsyg.2019.01282)
Supplement: Supplementary file 3 [file Table_3.docx]

# Appendixes

Table 3. Items of teacher-student relationships

| Scale | Item | Loading | | |
| --- | --- | --- | --- | --- |
|  |  | Reading | Mathematics | Science |
| PISA 2012 teacher- student relationship items (OECD, 2012) | 1. Students get along well with most teachers. | 0.74 | 0.71 | 0.75 |
|  | 2. Most teachers are interested in students’ well-being. | 0.72 | 0.73 | 0.78 |
|  | 3. Most of my teachers really listen to what I have to say. | 0.72 | 0.73 | 0.75 |
|  | 4. If I need extra help, I will receive it from my teachers. | 0.70 | 0.71 | 0.72 |
|  | 5. Most of my teachers treat me fairly. | 0.68 | 0.51 | 0.68 |
